# Danggui Buxue decoction regulates the immune function and intestinal microbiota of cyclophosphamide induced immunosuppressed mice

**DOI:** 10.3389/fphar.2024.1420411

**Published:** 2024-08-19

**Authors:** Huan Huang, Yufei Xie, Xifeng Li, Fuxing Gui, Pingrui Yang, Yutao Li, Li Zhang, Hongxu Du, Shicheng Bi, Liting Cao

**Affiliations:** ^1^ Department of Traditional Chinese Veterinary Medicine, College of Veterinary Medicine, Southwest University, Chongqing, China; ^2^ Weifang Academy of Agricultural Sciences, Institute of Animal Husbandry, Shandong, China; ^3^ Hanzhong Animal Disease Prevention and Control Center, Shaanxi, China; ^4^ Chi Institute of Traditional Chinese Veterinary Medicine, Southwest University, Chongqing, China

**Keywords:** Danggui buxue decoction, immunosuppression, intestinal mucosal immunity, gut microbiota, immune function

## Abstract

**Ethnopharmacological relevance:**

Danggui Buxue decoction (DBD) is a traditional Chinese herbal formula. According to the theory of traditional Chinese medicine, the combination of *Astragali Radix* (AR) and *Angelica sinensis* (AS) is a classic prescription of tonifying *qi* and enriching blood. DBD has the functions of hematopoietic, immune enhancement and inflammation inhibition, usually used to treat qi and blood deficiency symptoms.

**Aim of the study:**

Cyclophosphamide (CY) can inhibit humoral and cellular immunity, leading to the overall immune disorder of the body, resulting in immunosuppressive (IS). Pre-laboratory studies confirmed the immunomodulatory effects of DBD, but its mechanisms have not been thoroughly studied. In this study, the main purpose was to determine the effects of DBD on the immune function and intestinal mucosal barrier function of IS mice induced by CY, and initially explored the immunomodulatory mechanism of DBD.

**Materials and methods:**

100 g of AR and 20 g of AS were accurately weighed and 0.5 g/mL of the DBD was obtained by boiling, filtration and rotary evaporation. Then, mice in the DBD group were administered 5 g/kg of DBD by gavage, positive group were administered 40 mg/kg of levamisole hydrochloride, whereas those in the control and model groups were given the corresponding volume of normal saline by gavage for 1 week. At the end of the experiment, blood, spleen, thymus, ileum and cecum contents of all the experimental mice were collected aseptically. IS mouse model induced by intraperitoneal injection of 80 mg/kg CY for three consecutive days. Pathomorphology was used to observe the physical barrier of the intestine, flow cytometry to detect splenic lymphocytes, immunohistochemistry to determine the content of intestinal barrier-associated proteins, ELISA to measure the secretion of ileal SIgA, qRT-PCR to detect the mRNA expression of immune-related genes in the intestine, and high-throughput sequencing and analysis of cecum contents.

**Results:**

DBD alleviated spleen tissue damage and restored impaired immune functions, such as increased thymus index and CD4+/CD8+ subsets of spleen lymphocytes. In addition, DBD could increase ileum villi length and the ratio of villi length to crypt depth (V/C), and decrease crypt depth. Moreover, DBD administration up-regulated the expression of ZO-1, Occludin, Claudin-1, MUC-2 mRNA in ileum. And the secretions of sIgA and ZO-1 in ileum were also significantly improved. Furthermore, the administration of DBD can increase the diversity of gut microbiota, improve the composition of intestinal flora and increase the relative abundance of beneficial genus, such as *Bacteroides*.

**Conclusion:**

DBD alleviated CY-induced immune damage by decreasing the ratio of spleen index to CD4+/CD8+ of T lymphocyte subsets. And the intestinal barrier function of mice was by improves improving the intestinal morphology of the ileum and up-regulating the expression levels of ZO-1, MUC-2 and SIgA. DBD regulates CY-induced gut microbiota dysregulation in mice by increasing species diversity and richness, regulating the phylum, class and order levels of *Bacteroidetes*.

## 1 Introduction

Host defense is one of the most basic functions of organisms, protecting humans and animals from infectious diseases (Yu et al., 2021; Sudeep et al., 2023). However, due to the damage of the immune system composed of cells, tissues and organs involved in the immune response, an immune abnormal state of the body’s ability to respond to antigens is called immunosuppression (Feng et al., 2021). Immunosuppression can lead to the gradual decline of the body’s immune capacity, weakened drug response, and more susceptibly infected by pathogenic microorganisms, which is difficult to treat, resulting in increased morbidity and mortality (Han et al., 2023; Yao et al., 2023a). Therefore, it is of great significance to search for safe immunomodulators to alleviate immunosuppressive diseases.

Cyclophosphamide (CY) is an active alkylating agent that is clinically used to treat diseases such as leukemia (Kumar and Venkatesh, 2016). However, CY can inhibit humoral and cellular immunity, leading to the overall immune disorder of the body, resulting in immunosuppressive side effects. Therefore, CY is commonly used in modern research to establish immunosuppressive animal models (Cui et al., 2022a; Nam et al., 2022; Nan et al., 2023). Intraperitoneal injection of CY induced weight loss, decreased immune organ index, cellular immune factors, immunoglobulin and immune cell activity in mice. CY also altered the gut microbiota composition and damaged the intestinal mucosa in mice (Huang et al., 2021; Zhang et al., 2023).

Chinese herbal medicine has been widely concerned for its safety, accessibility, anti-inflammatory and immunomodulatory effects. According to deficiency theory of TCM and earlier reports, long-term deficiency of qi and blood will lead to immunosuppression (Pan et al., 2023), so according to the principle of Chinese medicine treatment for immunosuppression diseases, the method of supplementing *qi* and blood should be adopted. Danggui Buxue decoction (DBD) first recorded in the book “*Neiwaishang Bianhuo Lun*” written by Li Dongyuan in the *Jin* and *Yuan* dynasties, it is a famous prescription for supplementing *qi* and blood (Li et al., 2021; Zhou et al., 2022). It is composed of Astragalus mongholicus Bunge (Astragali Radix, AR) and Angelica sinensis (Oliv.) (Angelica sinensis, AS) (The plant name has been checked with http://www.worldfloraonline.org) (Liu et al., 2021a; Wang et al., 2022) with a ratio of 5:1. AR has the effect of tonifying *qi* and strengthening superficial resistance of the body, promoting diuresis, detoxifying, discharging pus, astringing sores and promoting tissue regeneration. AS has the effect of enriching blood and activating blood circulation, regulating menstruation to relieve pain, moistening bowels and relieving constipation. The combination of these two herbal medicines is a classic prescription of tonifying *qi* and enriching blood. Modern research believes that *qi* is closely related to immunity. In traditional Chinese medicine syndrome differentiation and treatment, immune deficiency belongs to asthenic syndrome, that is, lack of vital *qi* will lead to the invasion of evil *qi*. Modern pharmacological studies have confirmed that DBD has the functions of hematopoietic, immune enhancement and inflammation inhibition (Kwan et al., 2021). At present, the pharmacological effects of DBD are mainly in its promotion of hematopoietic and anti-tumor aspects (Liu et al., 2021b; Xiang et al., 2023), while the studies on the regulation of animal immunity are relatively few. Previous laboratory studies discussed the effect of different doses of DBD on the immunity of type O foot-and-mouth disease vaccine and intestinal mucosal immunity. The results showed that 5 g/kg DBD had the best effect (Zhou et al., 2022). However, the immunomodulatory effects of DBD and its mechanisms have not been thoroughly studied, especially the immune-protective mechanisms under the state of immunosuppression is still unclear.

Therefore, the main purpose of this study was to investigate the immunomodulatory effect and mechanism of DBD by CY induced IS mouse model. We used pathological examination, ELISA, molecular biology techniques, high-throughput sequencing and other methods to determine the effects of DBD on the immune function and intestinal mucosal barrier function of IS mice, and initially explored the immunomodulatory mechanism of DBD. We believe that this study not only helps to reveal the immunomodulatory mechanism of DBD, but also provides a lot of scientific evidence for the development of new immune enhancers.

## 2 Materials and methods

### 2.1 Drugs and reagents

*Astragali radix* [Fabaceae; Astragalus mongholicus Bunge](210303) was purchased from Gansu Guocao Pharmaceutical Co., LTD.; *Angelica sinensis* [Apiaceae; Angelica sinensis (Oliv.)] (201201) was purchased from Chongqing Yafeng Pharmaceutical Co., LTD.; Cyclophosphamide (C849559) and levamisole hydrochloride (L812625) were purchased from Shanghai Maclin Biochemical Technology Co., LTD.; Rat Anti-Mouse CD3ε-APC (1535-11), Rat Anti-Mouse CD4-FITC (1540-02s), and Rat Anti-Mouse CD8-PE (1550-09) were purchased from Southern United States Biotech Inc.; Mouse spleen lymphocyte isolation kit (LTS1092PK), purchased from Tianjin Hao Yang Biological Products Technology Co., LTD. HRP-Goat Anti-Rabbit IgG (ZB-2301) was purchased from Zhongshan Jinqiao. Rabbit Anti-ZO-1 antibody (bs-1329R) from Beijing Boosen Biotechnology Co., LTD. Mouse SIgA Elisa kit (BPE20416) was purchased from Shanghai Langton Biotechnology Co., LTD. RNAiso Plus (9109), PrimeScript RT reagent kit with gDNA eraser (RR047A), TB Green premix ex taq II (Tli RNaseH Plus) (RR820A), Purchased from Baori Doctor Physical Technology (Beijing) Co., LTD.; Mouse ATCB internal reference primer (B661302-0001), purchased from Bioengineering (Shanghai) Co., LTD.

### 2.2 Animal and experimental design

Forty six-week-old specific pathogen-free (SPF) male Kunming mice with a body weight of 26 ± 2 g were purchased from Sibeifu (Beijing) Biotechnology Co., LTD (Animal license NO. SCXK [Beijing] 2019-0010). The animals were housed at a temperature of 22°C ± 2°C and relative humidity of 55% ± 10%, subjected to a 12-h/12-h light/dark cycle, and provided access to food and water *ad libitum* for 1 week. All animal experiments were performed with approval by the Laboratory Animal Ethics Committee of Southwest University (permit number: IACUC-20211020-01). And all the experimental animals were euthanized at the end of the experiment.

### 2.3 Preparation of DBD

DBD was prepared according to the method previously obtained (Zhou et al., 2022). In short, accurately weigh 100 g of AR and 20 g of AS, soak in 8 times distilled water at high temperature for 30 min to boil, and then gently simmer for 1 h. Strain the liquid and add the residue to an equal amount of distilled water for another 1 h. The two extracts were mixed and concentrated to obtain a crude drug content of 0.5 g/mL using a rotary evaporator.

### 2.4 Animal modeling and drug administration

After adaptive feeding, 40 mice were randomly divided into control group, model group, positive group and DBD group, with 10 mice per group. Mice in the model, positive and DBD groups were intraperitoneally injected with 80 mg/kg CY for 3 consecutive days to establish the IS model. Then, mice in the DBD group were administered 5 g/kg of DBD by gavage, positive group were administered 40 mg/kg of levamisole hydrochloride, whereas those in the control and model groups were given the corresponding volume of normal saline by gavage for 1 week. At the end of the experiment, blood, spleen, thymus, ileum and cecum contents of all the experimental mice were collected aseptically.

### 2.5 Body weight and immune organ index

Each mouse was weighed daily. And at the end of the experiment, spleen and thymus of all mice were collected aseptically and weighed. The organ index is calculated as follows:

Organ index = organ weight (mg)/mouse weight (g).

### 2.6 Flow cytometry

The spleen was ground with a syringe piston and passed through a 70 μm cell screen to form a cell suspension. The cells were gently washed twice in an ice bath with PBS to adjust the cell concentration to 1×10^6^/mL. CD3-APC, CD4-FITC, and CD8-PE were added with 1 μL each, and incubated on ice for 30 min in dark light, then suspended twice with 1 mL PBS, centrifuged (2500 rpm, 5 min), abandoned the supernatant, and added 800 μL PBS to blow and mixed well. Finally, lymphocytes were detected by flow cytometry.

### 2.7 Hematoxylin-eosin staining (HE)

The ileum tissue was fixed in 4% neutral formaldehyde, trimmed to a suitable size, washed with running water overnight, dehydrated with ethanol gradient, and embedded in paraffin. The paraffin-embedded ileum was cut into 5 μm sections for HE staining, and the villi length and crypt depth were measured by ImageJ software. The ratio of villi length to crypt depth was calculated.

### 2.8 Immunohistochemistry staining (IHC)

The paraffin-embedded ileum was cut into 5 μm slices and fixed on adhesive slides. After gradient dehydration with ethanol, endogenous peroxidase was removed with 3% hydrogen peroxide, and antigen repair was performed in sodium citrate buffer solution. The ileum was closed with 5%BSA for 30 min and incubated overnight with Rabbit Anti-ZO-1 antibody at 4°C. On the second day, Goat Anti-Rabbit IgG droplets labeled by HRP were added to the slide, stained by DAB and HE, and observed by microscope.

### 2.9 Enzyme-linked immunosorbent assay (ELISA)

Mouse ileum tissue was homogenized, supernatant was obtained by centrifugation, and SIgA content was detected according to manufacturer’s instructions.

### 2.10 Real-time reverse transcription–polymerase chain reaction (qRT-PCR)

Total ileum RNA was extracted by TRIZOL method and reversed-converted into cDNA. mRNA expression levels of ZO-1, Claudin-1, Occludin, MUC-2 and IgA in ileum tissue were detected by real-time fluorescence quantitative PCR. Using *β-actin* as internal reference gene, the primer sequences are shown in Table 1 and the data were analyzed using 2^−△△ct^ method.

**TABLE 1:** Primer sequence.

Gene name	NCBI (entry number)	Primer sequence	Product length (bp)
ZO-1	NM_001163574.1	F: AACCCGAAACTGATGCTGTGGATAG R: CGCCCTTGGAATGTATGTGGAGAG	150
Claudin-1	NM_016674.4	F: GCTGGGTTTCATCCTGGCTTCTC R: CCTGAGCGGTCACGATGTTGTC	110
Occludin	NM_001360536.1	F: TGGCTATGGAGGCGGCTATGG R: ACTAAGGAAGCGATGAAGCAGAAGG	117
MUC-2	NM_023566.4	F: CGAGCACATCACCTACCACATCATC R: TCCAGAATCCAGCCAGCCAGTC	95
IgA	NM_007655.4	F: GCCCTGCCTCTCCTCCTCTTC R: CTCGCCCAAGTTCACCGTCAG	105

### 2.11 16S rRNA sequencing of gut microbiota

Gut microbiota community gene DNA was extracted from mouse cecum contents according to the instructions of fecal DNA extraction kit. Universal primers 338F (5′-ACT​CCT​ACG​GGA​GGC​AGC​AG-3′) and 806R (5′-GGACTACHVGGGTWTCTAAT-3′) were used to amplify the V3-V4 region of bacterial 16s rRNA gene. The PCR products were sequenced and analyzed on a high-throughput sequencer. The sequencing results were filtered and spliced (Pear software), and the sequences with 97% similarity were clustered into operation classification units (OTU). The sequences were compared with Silva138 database (Silva138), and R software was used for mapping.

### 2.12 Statistical analysis

All data were expressed as the mean ± standard error (SE) and analyzed using GraphPad Prism 8. Statistical significance was determined using IBM SPPS 22.0, and differences were considered significant at *p* < 0.05.

## 3 Results

### 3.1 Evaluation of the IS mice model

**FIGURE 1:**
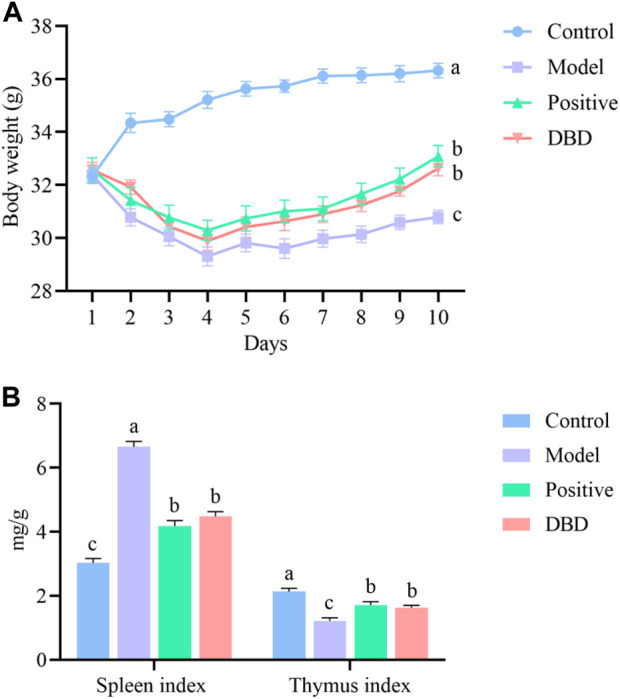
Evaluation of the IS mice model. **(A)** body weight; **(B)** organ index.

The status and weight changes of mice before and after CY injection were recorded to evaluate whether the model was successfully established. Compared with the control group, the mice injected with CY showed symptoms such as lethargy. After CY injection, the mice body weight began to decrease linear and significantly different compared with that before the injection (Figure 1A). Moreover, the thymus index significantly decreased and the spleen index significantly increased (Figure 1B) in the model group compared with the control group (*p* < 0.05). These results indicated that the IS mice model was successfully established.

### 3.2 Effect of DBD lymphocyte subsets of spleen in IS mice

**FIGURE 2:**
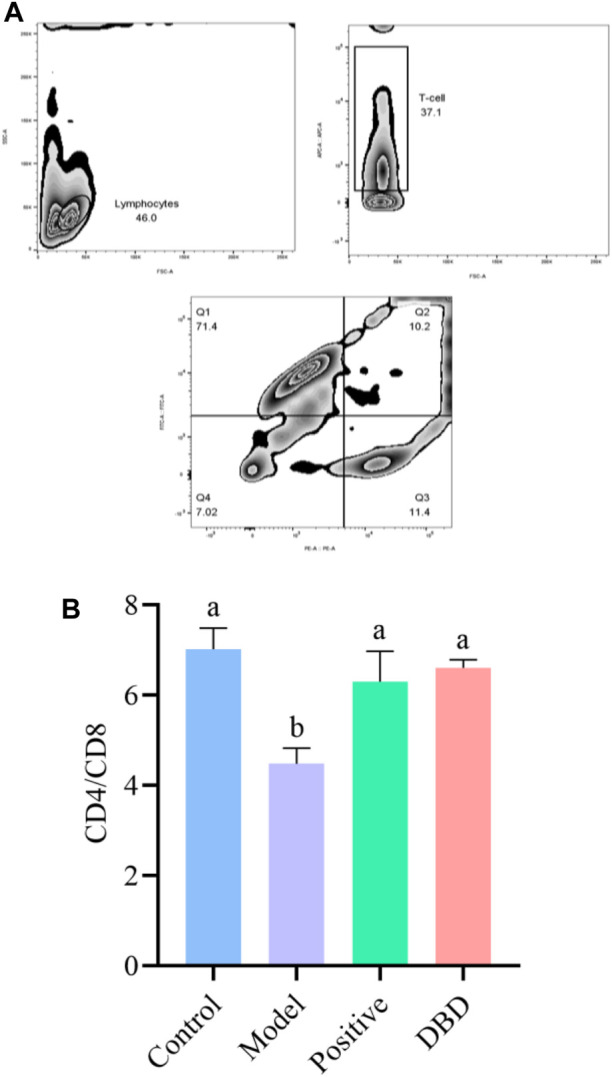
Effect of DBD on lymphocyte subsets of spleen in IS mice. **(A)** zebra plot of flow cytometry; **(B)** CD4+/CD8+ subsets.

As shown in Figure 2A, the distribution analysis of splenic lymphocyte subsets of mice in each group showed that splenic lymphocyte, CD3^+^ cells and CD4+/CD8+ T cells were obviously grouped. According to Figure 2B, the CD4+/CD8+ ratio of spleen lymphocytes in the model group was significantly lower than that in the control group, positive group and DBD group (*p* < 0.05).

### 3.3 Effect of DBD on the morphology of ileum in IS mice

**FIGURE 3:**
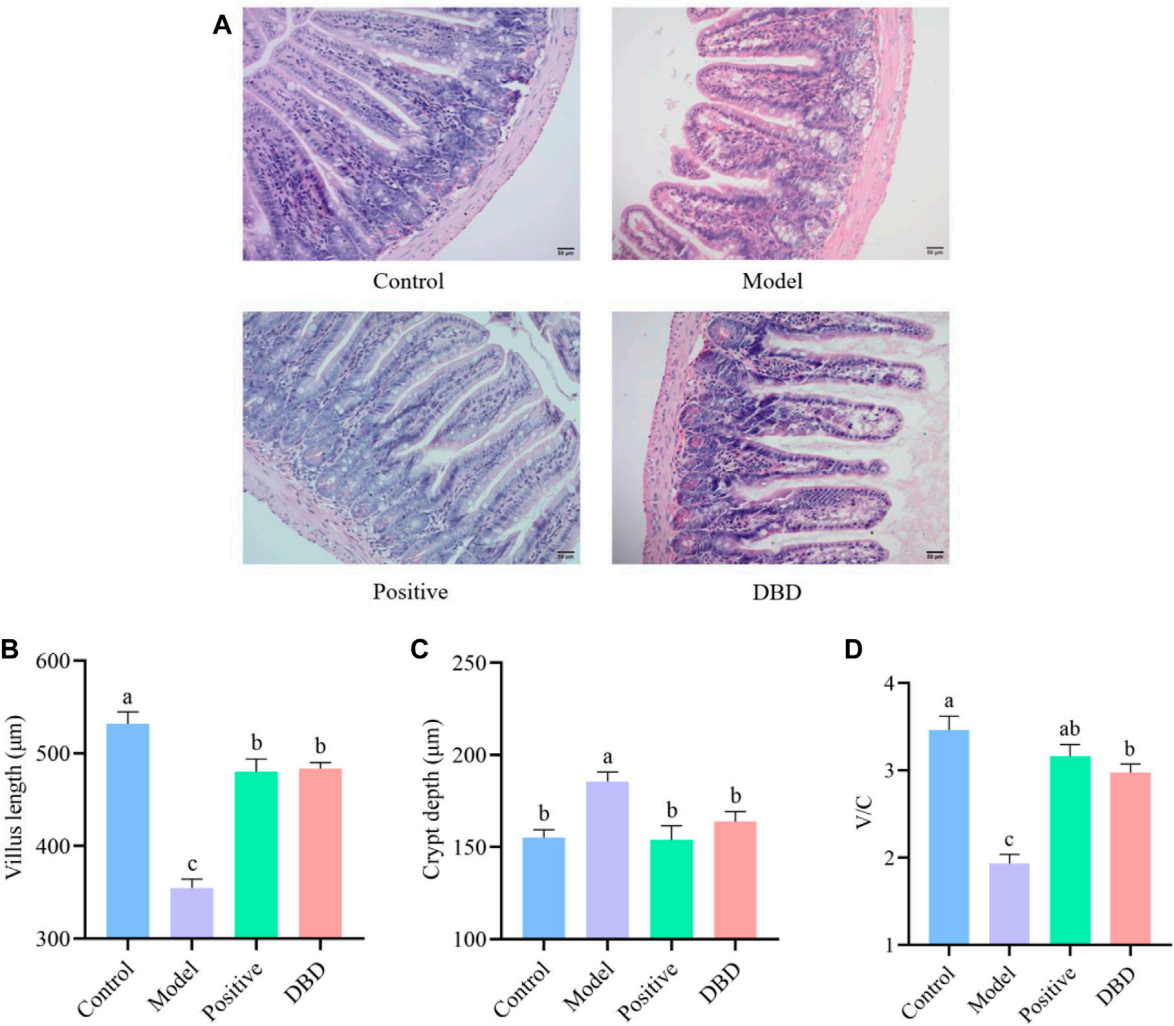
Effect of DBD on ileum morphology in IS mice. **(A)** HE; **(B)** Villus length; **(C)** Crypt depth; **(D)** V/C.

HE staining was used to observe the morphological and structural changes of the ileum tissue of experimental mice in each group, and the test results were shown in Figure 3. Compared with the control group, the villi length of was shorter, the intestinal recess was deeper and the ratio of V/C was significantly decreased in model group (*p* < 0.05). However, compared with the model group, the depth of crypt was decreased, villus length and V/C was increased in DBD group (*p* < 0.05).

### 3.4 Effect of DBD on intestinal mucosal barrier in IS mice

**FIGURE 4:**
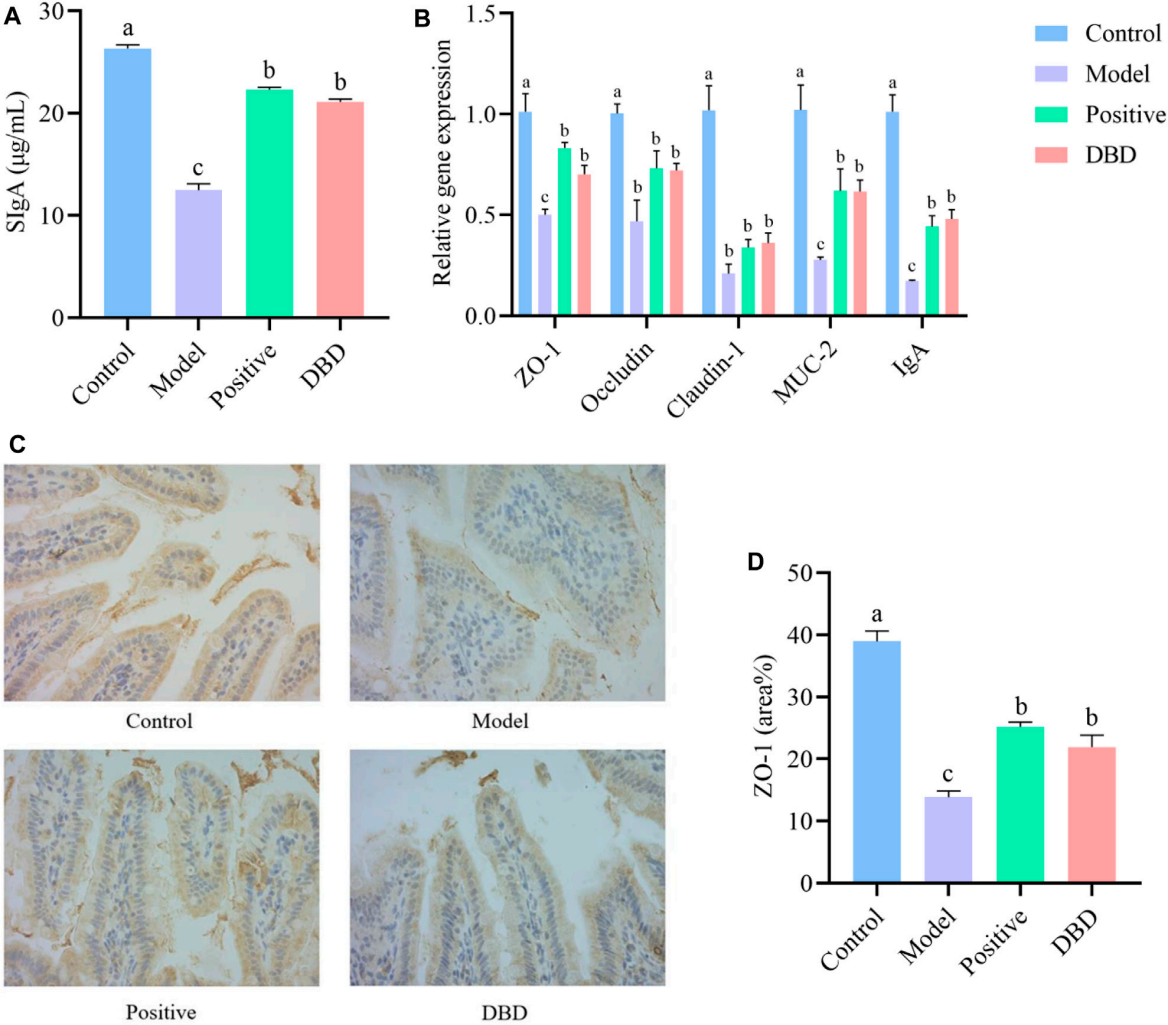
Effect of DBD on intestinal mucosal barrier in IS mice. **(A)** SIgA secretion; **(B)** mRNA expression levels; **(C)** IHC; **(D)** ZO-1 protein area.

We evaluated the effects of DBD on intestinal mucosal barrier function in IS mice by detecting physical barrier, chemical barrier and immune barrier. Compared with the control group, the SIgA secretion was significantly reduced in model group, while SIgA secretion in ileum was improved after DBD treatment (Figure 4A). As shown in Figure 4B, CY can significantly downregulate mRNA expression levels of ZO-1, Occludin, Claudin-1, MUC-2 and IgA (*p* < 0.05). After DBD treatment, the mRNA expression levels of ZO-1, MUC-2 and IgA significantly upregulated (*p* < 0.05). The results of IHC (Figures 4C, D) were similar to those of qRT-PCR. Compared with control group, CY decreased the expression level of ileum ZO-1 protein and increased intestinal permeability, while DBD improved intestinal permeability (*p* < 0.05).

### 3.5 Effect of DBD on gut microbiota structure in IS mice

**FIGURE 5:**
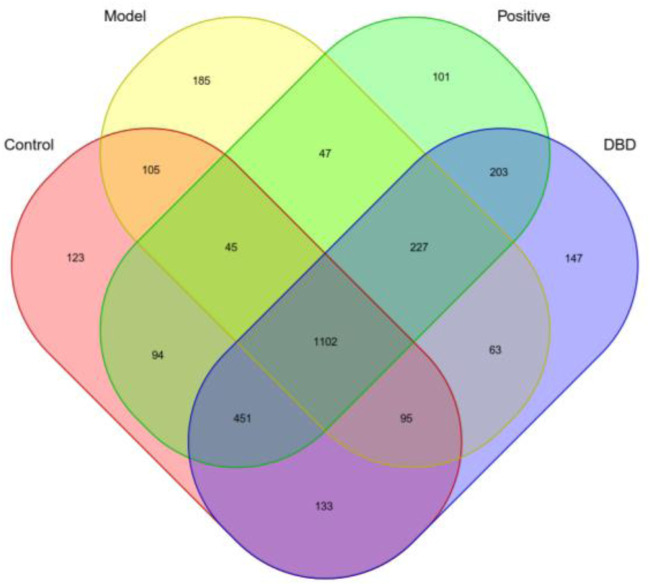
Veen diagram of mice gut microbiome OTU.

As shown in Figure 5, a total of 3121 OTU were obtained, including 1102 shared OTU in the four groups. There were 1347, 1692, 1781 OTU in control group compared with model group, positive group and DBD group, respectively. The number of unique OTU in model group was the highest, but the positive group and DBD group was similar to control group.

### 3.6 Analysis of alpha diversity of gut microbiota in IS mice by DBD

**FIGURE 6:**
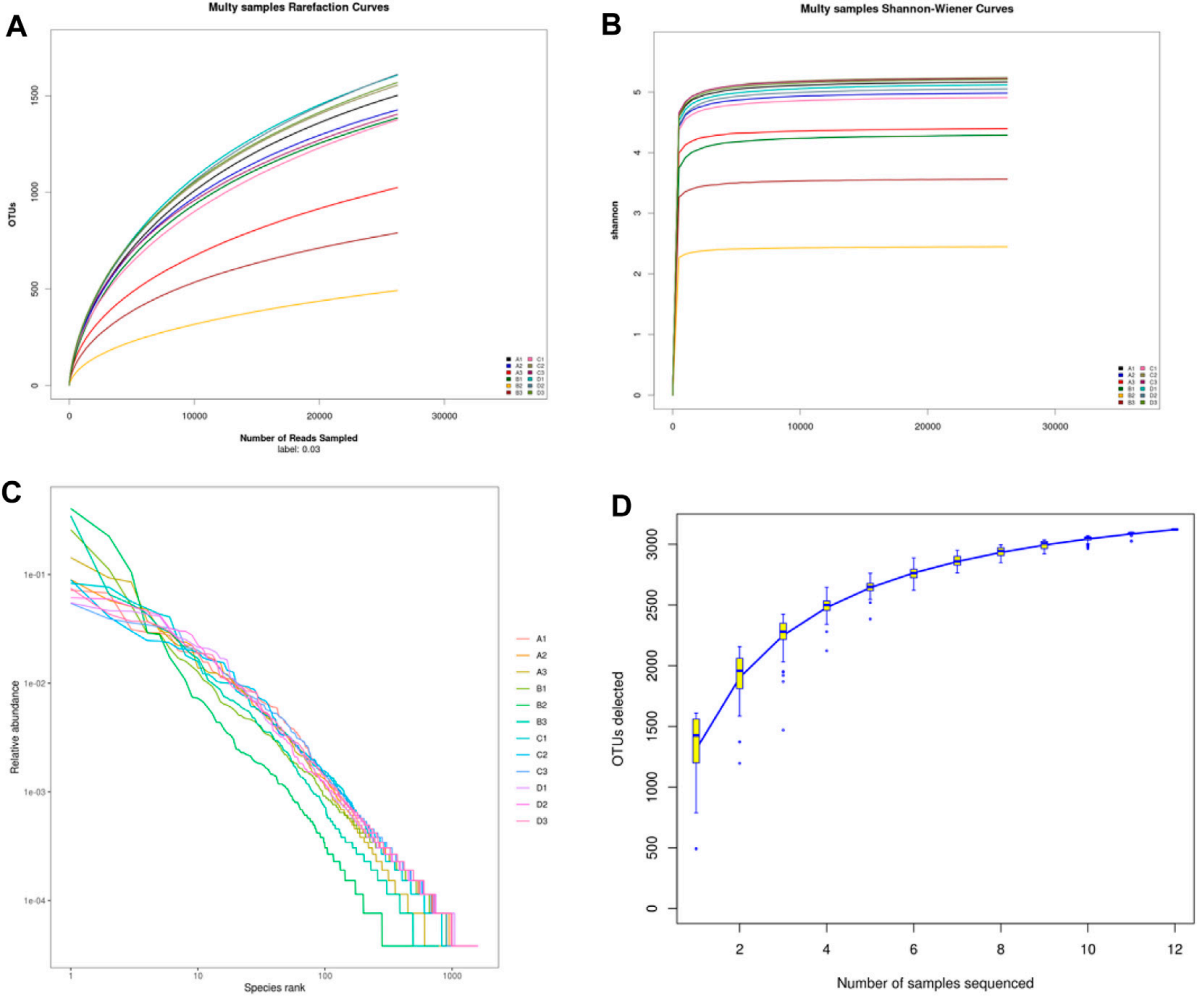
Alpha diversity curve. **(A)** Multy samples rarefaction curves; **(B)** Multy samples Shannon-wiener curves; **(C)** Rank abundance curve; **(D)** Specaccum accumulation curve.

**FIGURE 7:**
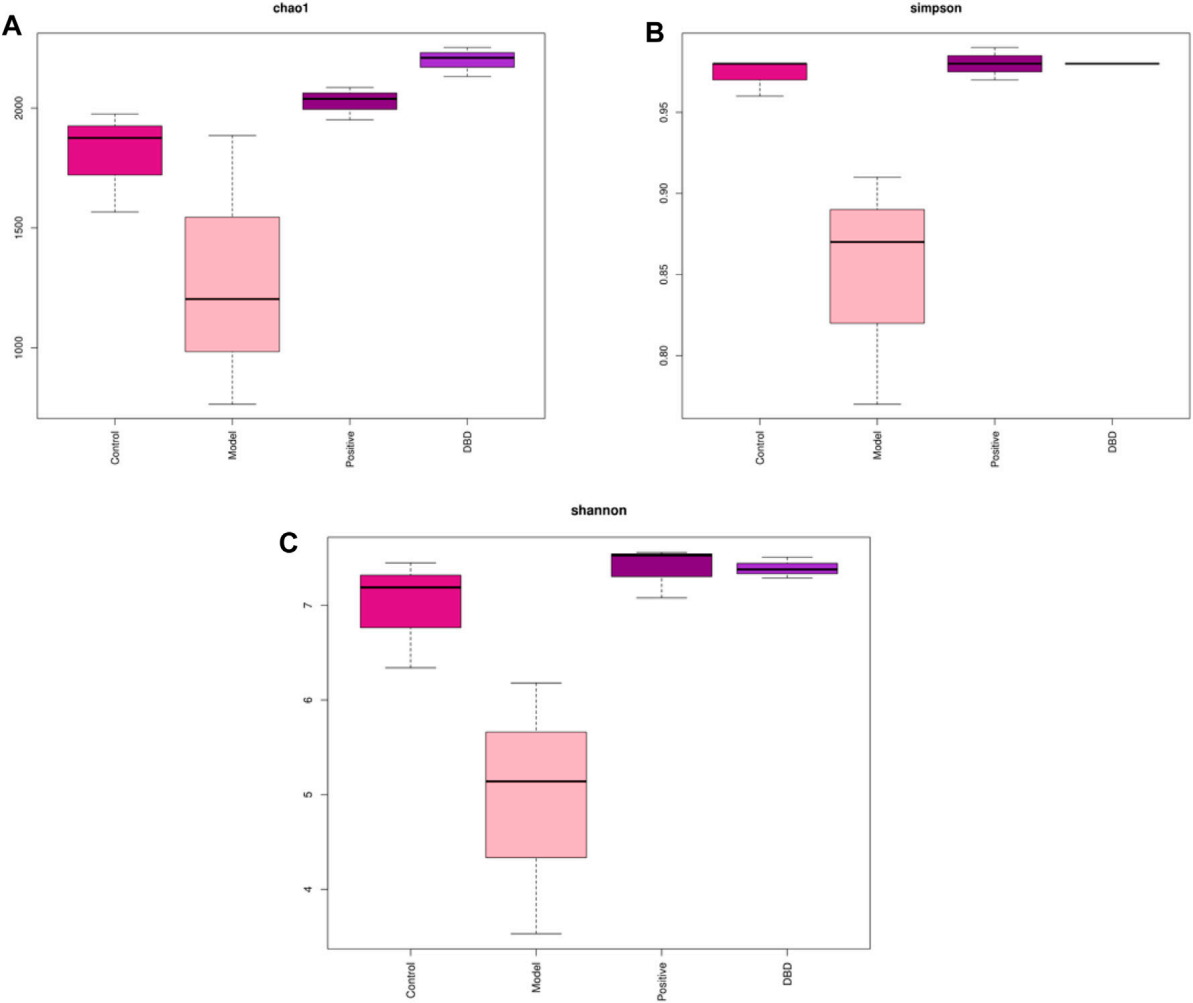
Index of Alpha diversity. **(A)** Chao 1; **(B)** Simpson; **(C)** Shannon.

Alpha diversity was mainly used to analyze the diversity of gut microbiota among each group. As shown in Figure 6, the dilution curve (Figure 6A), Shannon index curve (Figure 6B), class abundance curve (Figure 6C) and species accumulation curve (Figure 6D) tend to be flat, indicating sufficient sequencing data and high species richness and evenness. Compared with the control group, the indexes of Chao 1 (Figure 7A), Simpson (Figure 7B) and Shannon (Figure 7C) in the model group was decreased, among which Simpson and Shannon indexes decreased significantly (*p* < 0.05). This indicating that the species abundance and diversity of cecal colonies decreased. Compared with the model group, the indexes of Chao 1, Simpson and Shannon of mice in positive group and DBD group were significantly increased (*p* < 0.05). This indicates that DBD could effectively alleviate the decline in cecum colony abundance and diversity caused by CY.

### 3.7 Analysis of beta diversity of gut microbiota in IS mice by DBD

**FIGURE 8:**
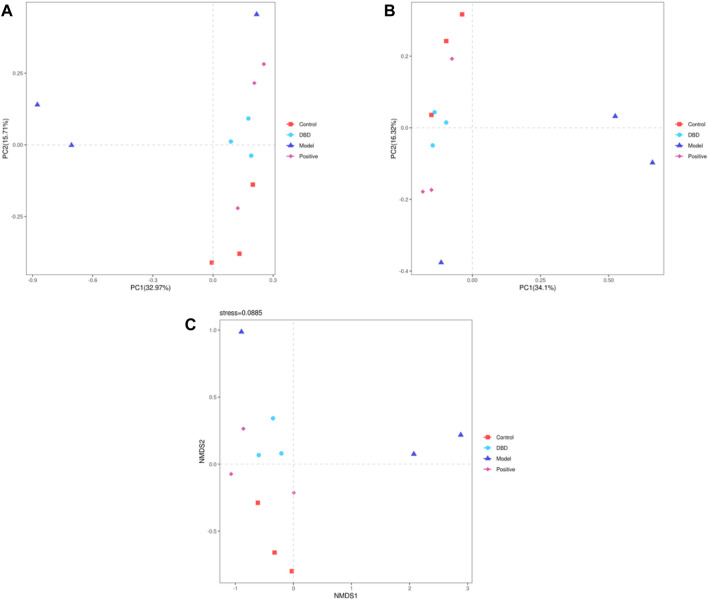
Beta diversity analysis. **(A)** PCA; **(B)** PCoA; **(C)** NMDS.

The Beta diversity index further analyzed the effects of CY treatment on the richness and diversity of intestinal microbiota in mice. PCA (Figure 8A) and PCoA (Figure 8B) analyses are distance-based Beta diversity analyses, that is, the closer the distance between samples, the more similar the sample composition. As shown in Figures 8A, B, within a certain range, PCA and PCoA maps showed that the microbial composition of each group was similar, and the microbial composition of each group was clustering. In general, the distance between the model and control, positive and DBD group was relatively far, means the differences were obvious. NMDS analysis (Figure 8C) were consistent with those of PCA and PCoA. These results showed that DBD could improve the changes of gut microbiota in mice induced by CY.

### 3.8 Effect of DBD on gut microbiota composition of IS mice

In order to further analyze the potential protective mechanism of DBD on IS mice, 16s rRNA sequencing was used to analyze the effect of DBD on gut microbiota of IS mice. A total of 18 of phylum levels, 38 of class levels, 88 of order levels, 148 of family levels, 286 of genus levels and 202 of species levels were identified in this experiment.

**FIGURE 9:**
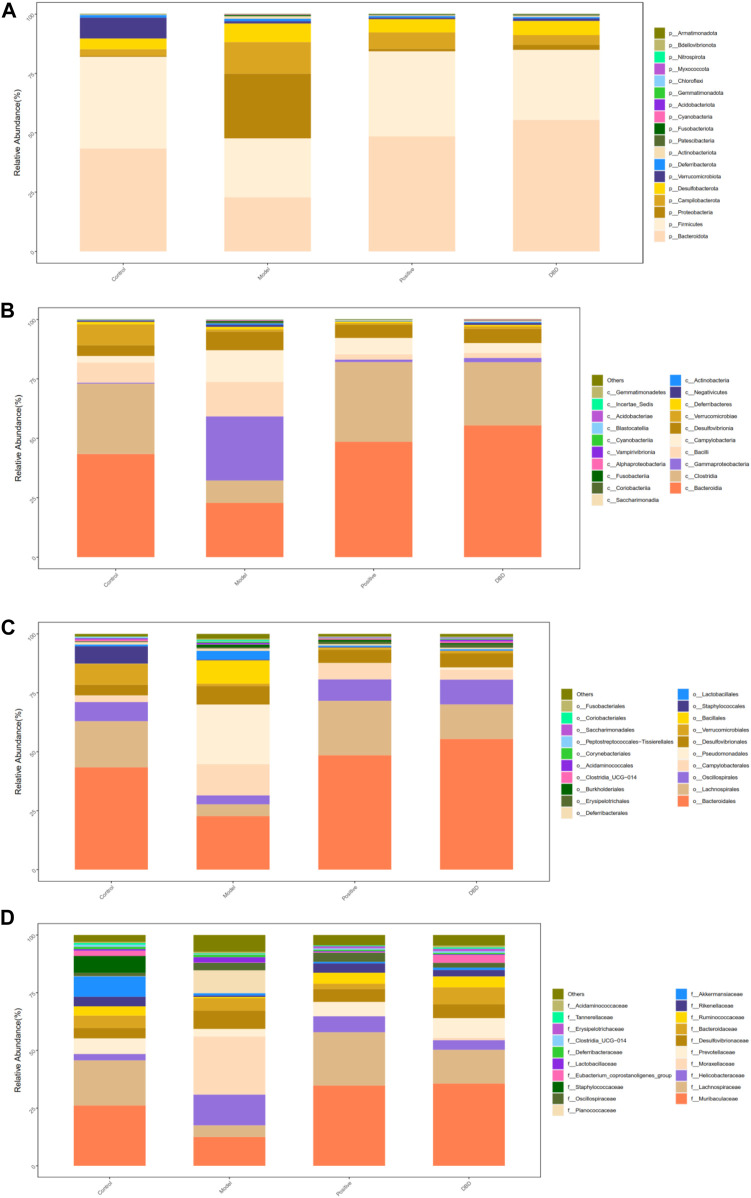
Gut microbiota composition. **(A)** phylum level; **(B)** class level; **(C)** order level; **(D)** family level.

At the phylum level (Figure 9A), the dominant bacteria are mainly composed of Firmicutes, Bacteroidota, Proteobacteria, Desulfobacterota and Campilobacterota. There was significant difference between the model group and other groups in Bacteroidota and Proteobacteria (*p* < 0.05). Firmicutes/Bacteroidota in the model group showed an increasing trend, but there was no significant difference compared with other group (*p* > 0.05). However, there was significant difference between the model group and the control group in Firmicutes level (*p* < 0.05).

At the class level (Figure 9B), Bacteroidia, Clostridia, Gammaproteobacteria, Bacilli and Campylobacteria are the main bacteroidia. There were significant differences between the model group and the other groups in Bacteroidia, Gammaproteobacteria and Clostridia (*p* < 0.05).

At the order level (Figure 9C), the dominant bacteria are mainly composed of Bacteroidales, Lachnospirales, Oscillospirales, Campylobacterales and Pseudomonadales. Compared with the control group, positive group and DBD group the model group had significantly differences in Bacteroidales and Pseudomonadales. There were significant differences in Lachnospirales level between the model group and the control group (*p* < 0.05).

At the family level (Figure 9D), Muribaculaceae, Lachnospiraceae, Helicobacteraceae, Moraxellaceae and Prevotellaceae are the main bacteroidia. Compared with control group, positive group and DBD group, the difference of model group in Moraxellaceae family level was statistically significant (*p* < 0.05). There was significant difference in Lachnospiraceae between the model group and the control group (*p* < 0.05).

### 3.9 Multivariate statistical analysis of DBD on IS mice

**FIGURE 10:**
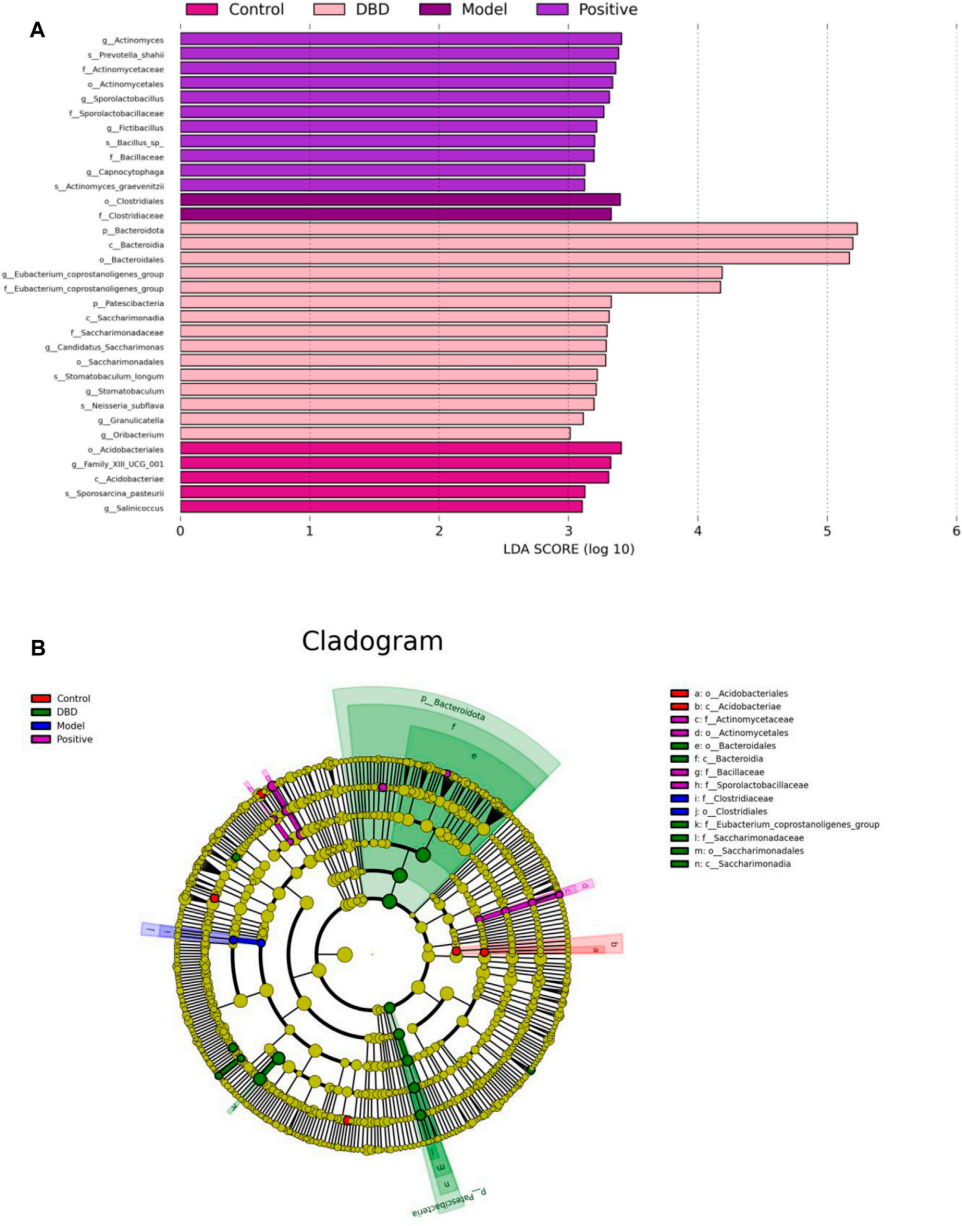
LEfSe statistical difference analysis. **(A)** LDA distribution bar graph; **(B)** LEfSe branching diagram.

The LDA distribution bar chart (Figure 10A) shows the species with LDA scores greater than the set value with differences, i.e., the biomaker with statistical differences. LEfSe analysis (Figure 10B) was used to detect different strains between groups. As can be seen from Figure 10A, a total of 33 taxonomic groups of different levels were identified in this experiment, and they had different degrees of richness in control group, model group, positive group and DBD group. Among them, in the control group, the main bacteria species were Acidobacteriales and Family_XIIIUCG_001. The model group was composed of Clostridiales and Clostridiaceae. The number of taxa in the positive group was the lowest, and the most significant species were *Actinomyces* and Prevotella_shahii. Bacteroidota, Bacteroidia and Bacteroidales were the most abundant species in the positive group. As can be seen from Figure 10B, Acidobacteriales and Acidobacteriae, Clostridiaceae and Clostridiales are significantly difference and play an important role in the control group and the model groups, respectively. The positive groups had four significant differences played an important role bacteroidia, there are Actinomycetaceae, Actinomycetales, Bacillaceae and Sporolactobacillaceae. And DBD groups were Bacteroidales, Bacteroidia, Saccharimonadaceae, Saccharimonadales, Saccharimonadia and Eubacterium_coprostanoligene.

## 4 Discussion

The immune system is a complex network of immune organs such as the spleen and thymus. As a natural barrier of the body, the immune system has the role of immune surveillance and defense. The intestinal barrier can eliminate the invasion of products and maintaining body health and homeostasis (Elmassry et al., 2022; Yan et al., 2022). Gut microbiota is a complex and changeable microcosm that inhabits the gut and participates in host immune regulation, protects the intestinal barrier and affects the functioning of the body. Many studies have shown that gut microbes are a key component of the gut barrier and play a key role in the development and regulation of immune function (Li et al., 2022; Yao et al., 2023b).

In this study, the weight and thymus index of CY-induced mice were decreased, which was consistent with the results of Chen et al. (Chen et al., 2021). It was proved that the CY induced IS model was successfully established in this experiment. Different from Liu et al. (Liu et al., 2022), this experiment found that spleen index of all experimental mice increased after injection of CY, which may be due to pathological enlargement of spleen caused by CY. The mechanism may be that the immunosuppressive effect of CY obstructs the development of thymus and the proliferation, differentiation and maturation of T lymphocytes (Xu et al., 2023). In the recovery period, the spleen showed obvious compensatory hyperplasia (Zhao et al., 2013). The ratio of CD4^+^ and CD8^+^ cells is one of the important indexes to evaluate the immune function of the body. The results of this experiment showed that intraperitoneal injection of CY in mice could cause the imbalance of T lymphocyte subsets in the animal body, resulting in cellular immune disorder, while DBD could increase the number of lymphocytes, and make CD4+/CD8+ close to normal by regulating the number of CD3^+^, CD4^+^ and CD8^+^ cells, so as to maintain the balance of immune function in the body.

CY is a widely used chemotherapy drug, and many studies have shown that it can damage intestinal mucosa (Chen et al., 2022). It can cause significant shortening of intestinal villi length, while crypt cells secrete a large number of immature cells in a short time when intestinal villi are destroyed, resulting in increased crypt depth (Liu et al., 2022). In this study, we confirmed that CY can cause villi damage in mice ileum tissue. The results of this study showed that DBD could improve the intestinal injury caused by CY to a certain extent, reduce the depth of crypts, increase the length of villi and V/C value, and make the intestinal mechanical barrier structure tend to be complete. Studies have shown that down-regulating the expression of ZO-1, Occludin and Claudin-1 can increase intestinal permeability and lead to enteric-related diseases (Al-Sadi et al., 2011; Lynn et al., 2020). The results of this study are similar, therefore, the improvement of intestinal physical barrier function by DBD may be related to the upregulation of mRNA and protein expression levels of ileum tight junction protein ZO-1. Previous studies have shown that MUC-2 is an important component of intestinal mucin layer (Okumura and Takeda, 2017; Cai et al., 2022), which can resist the invasion of enteroviruses, and its absence will affect mucosal repair. This explains the results of the model group of this experiment, CY can downregulate the expression level of intestinal MUC-2 mRNA, while DBD significantly upregulates the expression level of MUC-2 mRNA, indicating that DBD enhances the function of intestinal chemical barrier in mice to a certain extent. The SIgA results were consistent with the conclusion proved by Cai et al. (Cai et al., 2022) *in vivo* and *in vitro* experiments, which showed that spicosaccharide could enhance the secretion of SIgA to restore CY and lead to the decline of immune function. The results of this study showed that DBD increased the expression level of IgA and the concentration of SIgA in the ileum, thereby improving the function of intestinal immune barrier in IS mice.

Gut microbes are a key component of the gut barrier and play a key role in the development and regulation of immune function (Li et al., 2019; Tang et al., 2022). Intestinal microecological disturbance caused by changes in gut microbiota diversity, composition, structure and function will have a negative impact on the body and induce inappropriate immune activation. Therefore, the search for drugs that can regulate gut flora has important implications for body function. Many studies have shown that Chinese herbal medicines and their extracts have positive regulatory effects on the gut microbiota of animals (Pan et al., 2022; Wu et al., 2022). The high diversity of gut microbiota is conducive to the maintenance of intestinal stability. Alpha diversity analysis of intestinal species can reflect the richness and diversity of microbial community, and the decrease of richness and diversity is often associated with abnormal immune function (Tang et al., 2018). In this study, it was found that CY could lead to the decrease of Chao 1 index, Simpson index and Shannon index of cecal microflora in mice, which was similar to the results of Cui et al. (Cui et al., 2022a), that is, the diversity and richness of intestinal microflora in cecal contents decreased (Che et al., 2022; Cui et al., 2022b). At the phylum level, Firmicutes and *Bacteroides*, as the main dominant phyla, accounted for the highest relative abundance ratio in the gut microbiota detected in this study, and were the main components of the gut microbiota. Some of the bacteria in Firmicutes can enhance the immunity of the body by promoting the fermentation of polysaccharides and cellulose respectively. Bacteroidetes is one of the important groups of bacteria that maintain intestinal microbial homeostasis in animals. When Bacteroidetes change, its related effects will also change. An elevated ratio of Firmicutes to Bacteroidetes (F/B) is often considered a criterion for gut microbiota dysregulation. In this study, DBD can reduce the ratio of Firmicutes to Bacteroidetes, possibly by increasing Bacteroidetes and thereby decreasing F/B value. Bacteroidetes, as beneficial bacteria for intestinal colonization, plays an important role in inhibiting the proliferation of intestinal bacteria and producing short-chain fatty acids. Therefore, the increase of Bacteroidetes by DBD plays a beneficial role in the health of gut microbiota. At the family level, Lachnospiraceae can indirectly promote the production of short-chain fatty acids and play an anti-inflammatory and immunomodulatory role. The results of this study showed that compared with the blank control group, the content of Lachnospiraceae bacteria in the model group was significantly reduced. Studies have shown that Trichospirillaceae is one of the families that produce butyrate, which has many positive properties as it represents an important source of nutrients for intestinal cells, stimulates intestinal mucin production, improves tight junction protein integrity, and reduces inflammation. Thus, we speculate that CY may reduce butyrate production by decreasing Trichomillaceae, which leads to a decrease in inflammatory response as well as in mucin and tightenin.

The results of different levels of gut microbiota in mice showed that there were statistical differences between the model group and the control group, the positive group and the DBD group in Bacteroidota, Bacteroidia and Bacteroidales. According to the diversified statistical analysis, the significant differences in the groups of DBD were Bacteroidota, Bacteroidia and Bacteroidales. Therefore, we speculated that DBD may restore the balance of gut microbiota by regulating the level of phyla, class and order of *Bacteroides*.

## 5 Conclusion

**FIGURE 11:**
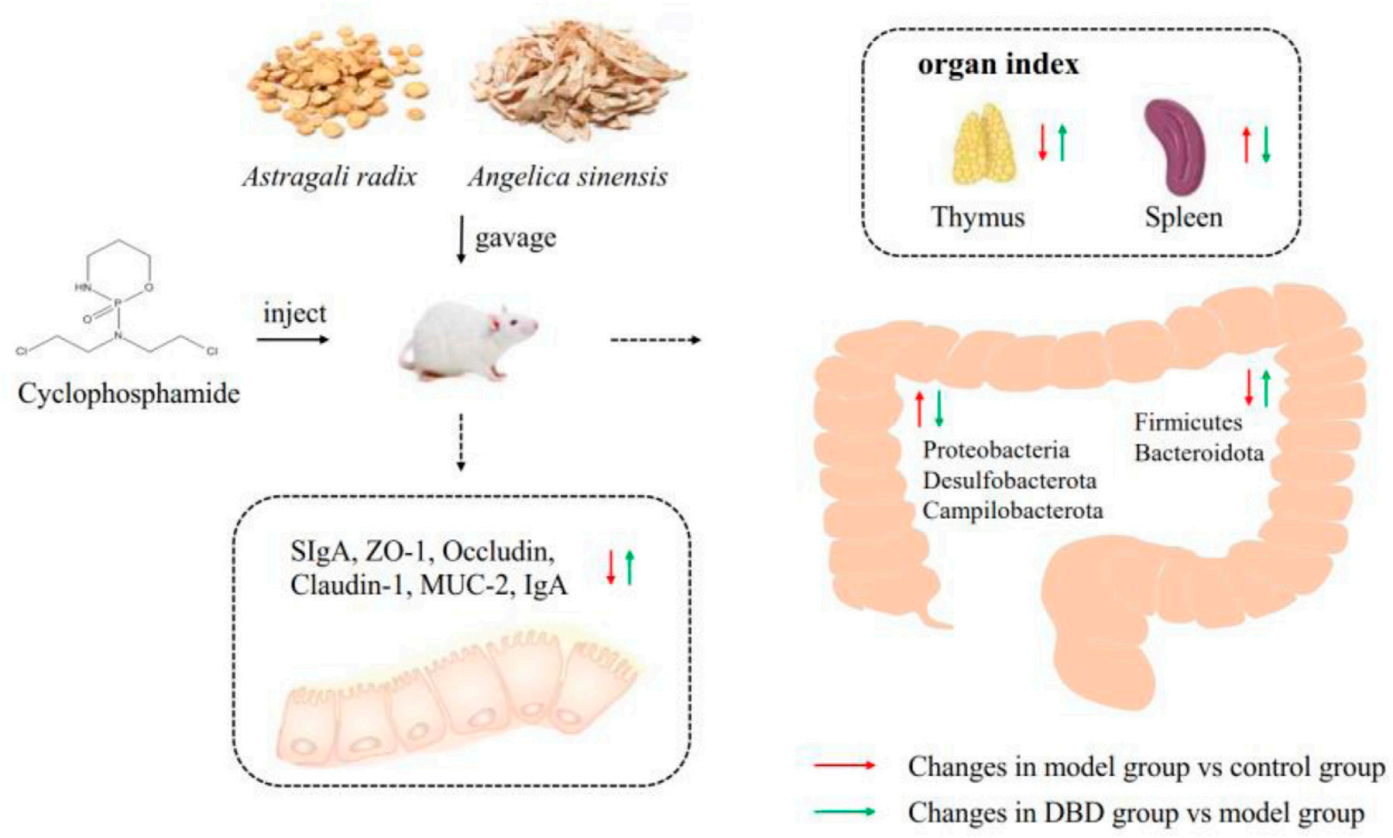
Summary diagram.

In this study, DBD alleviated CY-induced immune damage by increasing body weight and thymus index and decreasing the ratio of spleen index to CD4+/CD8+ of T lymphocyte subsets. And the intestinal barrier function of mice was by improves improving the intestinal morphology of the ileum and up-regulating the expression levels of ZO-1, MUC-2 and SIgA. DBD regulates CY-induced gut microbiota dysregulation in mice by increasing species diversity and richness, regulating the phylum, class and order levels of Bacteroidetes. In conclusion, DBD can protect gut health by regulating intestinal mucosal barrier and gut microbiota structure in mice (Figure 11).

## Data Availability

The data presented in the study are deposited in the NCBI repository, accession number PRJNA1147448.
